# Cerebral Venous Thrombosis in Two Patients with Spontaneous Intracranial Hypotension

**DOI:** 10.1155/2014/528268

**Published:** 2014-11-27

**Authors:** M. C. Garcia-Carreira, D. Cánovas Vergé, J. Branera, M. Zauner, J. Estela Herrero, E. Tió, G. Ribera Perpinyà

**Affiliations:** ^1^Department of Neurology, Hospital de Sabadell, Corporació Sanitària i Universitària Parc Taulí (CSIUPT), 08208 Sabadell, Spain; ^2^Department of Radiology, Hospital de Sabadell, Corporació Sanitària i Universitària Parc Taulí (CSIUPT), 08208 Sabadell, Spain

## Abstract

Although few patients with spontaneous intracranial hypotension develop cerebral venous thrombosis, the association between these two entities seems too common to be simply a coincidental finding. We describe two cases of spontaneous intracranial hypotension associated with cerebral venous thrombosis. In one case, extensive cerebral venous thrombosis involved the superior sagittal sinus and multiple cortical cerebral veins. In the other case, only a right frontoparietal cortical vein was involved. Several mechanisms could contribute to the development of cerebral venous thrombosis in spontaneous intracranial hypotension. When spontaneous intracranial hypotension and cerebral venous thrombosis occur together, it raises difficult practical questions about the treatment of these two conditions. In most reported cases, spontaneous intracranial hypotension was treated conservatively and cerebral venous thrombosis was treated with anticoagulation. However, we advocate aggressive treatment of the underlying cerebrospinal fluid leak.

## 1. Introduction

Spontaneous intracranial hypotension (SIH) indicates cerebrospinal fluid (CSF) leakage in the absence of a known dural puncture or tear. SIH is defined as CSF pressure ≤6 cm and/or imaging evidence of CSF leakage in patients with no history of lumbar puncture. SIH is characterized by the appearance of headaches or worsening of preexisting headaches within minutes of change in body posture from recumbent to standing position with improvement or even disappearance of pain on reclining [1].

Typical imaging features include subdural fluid collections that may mimic primary subdural hematomas, diffuse pachymeningeal gadolinium enhancement, engorgement of venous structures, pituitary hyperemia, sagging or downward displacement of the brain, and sometimes dilation of the vertebral venous plexuses with extradural fluid collections [2, 3].

Numerous cases of cerebral venous thrombosis (CVT) in patients with SIH have been reported since 2004 (see Table 1) [4–28]. We report two cases of patients with clinical signs of SIH who developed CVT.

## 2. Case Presentation

### 2.1. Case Report 1

A 29-year-old woman with history of migraine and no other known health problems presented with a 3-week history of headache. She was taking no medication other than oral contraceptives and did not smoke.

Head computed tomography (CT) ordered by her primary care physician was normal. With a presumptive diagnosis of sinusitis, she underwent 7 days' treatment with amoxicillin/clavulanic acid. General analgesics, nonsteroidal anti-inflammatory drugs, and benzodiazepines brought no relief.

She presented at our emergency department for persistent headaches. From the onset, her symptoms were orthostatic and disappeared on lying down. During this period, the headache was frontal and orbital, nonpulsating, with photophobia and phonophobia. No rhinorrhea, lacrimation, or conjunctival injection was present. The patient reported no prior dural puncture, surgical intervention, or trauma.

Findings at physical and neurological examinations, routine blood tests, and immunology were unremarkable.

**Figure 1:**
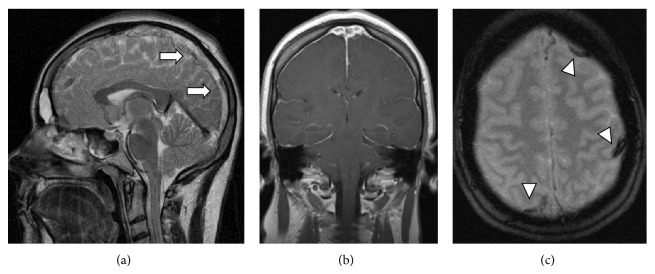
Brain MRI. (a) Sagittal T2-weighted image, (b) coronal T1-weighted image, and (c) axial gradient-echo image show superior sagittal sinus thrombosis (arrow) and thrombosis of multiple cortical cerebral veins (arrowheads).

Magnetic resonance imaging (MRI) (Figure 1) showed thrombosis of the superior sagittal sinus and of multiple cortical cerebral veins. There was no parenchymal damage. Treatment with intravenous heparin followed by oral anticoagulation did not improve the headaches.

Radionuclide cisternography revealed both direct and indirect signs of intracranial hypotension (delayed radiotracer ascent, with retained activity in the basal cisterns and no uptake in the cerebral convexity, together with early concentration of radiotracer in the bladder). Radionuclide cisternography also detected a slight left dorsal parameningeal uptake at the T4-T5 level that could correspond to a CSF leak. The CSF opening pressure was 3 cm H_2_O. The composition of the CSF was normal.

A dorsal epidural blood patch provided good but temporary relief from symptoms. Three weeks later, a second blood patch achieved complete resolution of symptoms within two weeks.

Two months after onset, with the patient asymptomatic and still on oral anticoagulants, MRI showed extensive but incomplete recanalization of the superior sagittal sinus; no signs of SIH were present.

Extensive investigation for thrombophilia was negative except for hyperhomocysteinemia. Oral contraceptives were not discontinued.

### 2.2. Case Report 2

A 54-year-old previously healthy man was admitted from another hospital for subarachnoid hemorrhage with left hemiparesis and hemihypesthesia.

During the previous two weeks, he had complained of a progressive headache and neck pain associated with dizziness. The headache was oppressive and holocranial, but particularly intense in the occipital region. It had strong postural variation, appearing only in the upright position and disappearing within seconds after lying down. He had no history of trauma. He developed numbness of the left limbs and fell, resulting in head injury and admission to the other hospital.

Physical examination detected a contused wound in the left temporal area. Neurological examination showed left hemiparesis with hemihypesthesia and left hemianopsia.

**Figure 2:**
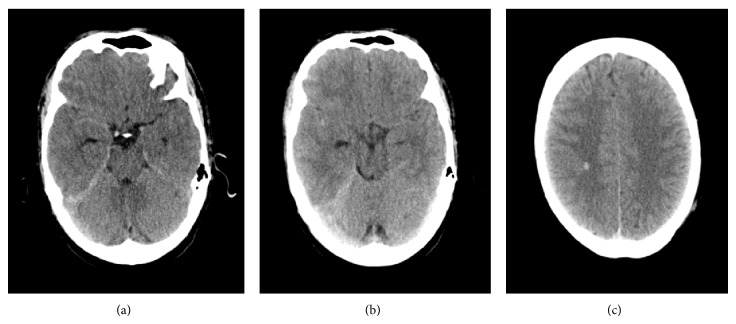
CT. ((a), (b)) Right frontoparietal subarachnoid hemorrhage. (c) Right parietal hematoma measuring 8 mm in diameter.

CT scan revealed right frontoparietal subarachnoid hemorrhage and a right parietal hematoma measuring 8 mm in diameter (reported as a probable venous malformation) (Figure 2). The patient was treated with nimodipine and transferred to our hospital for further tests.

Cranial CT angiography showed hypoplasia of the A1 segment of the right anterior cerebral artery. The carotid artery, vertebral artery, and other branches of the circle of Willis were of normal size and patency.

**Figure 3:**
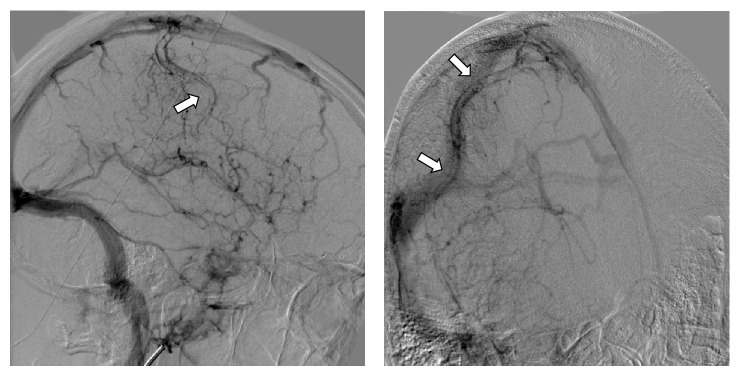
Cerebral angiography. Venous phase image of the right internal carotid artery shows a tubular filling defect within the superficial cortical vein of Trolard (arrow) and engorgement of the surrounding venules, suggestive of thrombosis of the vein of Trolard.

Digital subtraction angiography to investigate the cause of intracerebral hemorrhage (Figure 3) found no aneurysms or arteriovenous malformations but revealed a tubular filling defect within the right frontoparietal superficial cortical vein (vein of Trolard) and signs of congestion of the other deep and superficial veins of the brain. Contrast material was slow to empty from the left transverse sinus, suggesting moderate intracranial hypertension.

After treatment with subcutaneous heparin and subsequently with oral anticoagulants, the patient's neurological deficits resolved but his headaches persisted. The headache worsened when standing and improved shortly after lying down.

**Figure 4:**
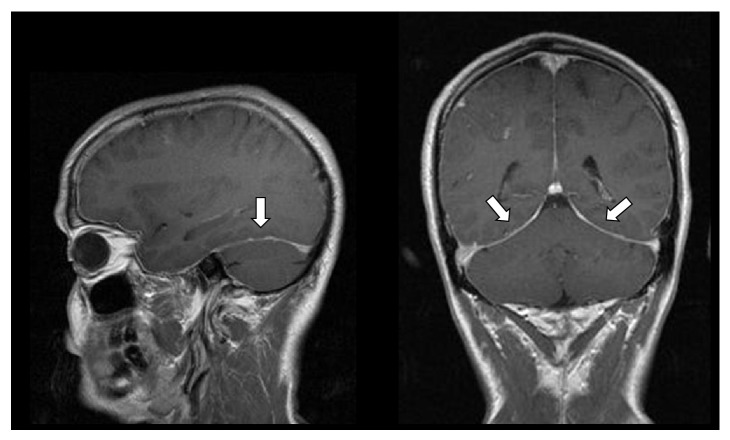
Brain MRI. Diffuse pachymeningeal enhancement after gadolinium administration (arrows), suggestive of liquoral hypotension.

Brain MRI (Figure 4) to investigate this headache showed diffuse pachymeningeal enhancement after gadolinium administration, suggestive of liquoral hypotension, and a small right parietal hematoma in the acute phase (correlating with the CT findings).

Radionuclide cisternography showed delayed ascent of radiotracer, with no uptake in the cerebral convexity, and retained activity in the basal cisterns, together with intense renal and bladder activity (indirect signs of intracranial hypotension). It was impossible to locate the level of the CSF leak with certainty, although it was thought that some parameningeal punctiform images immediately to the left of T3-T4 might correspond to a leak.

CSF opening pressure was 2 cm H_2_O; the composition of the CSF was normal.

The patient was diagnosed with intracranial hypotension associated with intraparenchymal hematoma secondary to cortical venous thrombosis. Treatment with oral anticoagulants and blood patch brought about complete resolution of symptoms.

## 3. Discussion

The diagnosis of SIH is based on a purely orthostatic headache (increased intensity when upright and decreased intensity when lying down) occurring in the absence of recent dural puncture or trauma. SIH can lead to nausea and vomiting. SIH may also produce traction on the cranial nerves, causing diplopia, hearing loss, tinnitus, dysgeusia, dizziness, visual deficits, and neck pain. SIH is confirmed by a CSF opening pressure of <6 cm H_2_O and/or evidence of CSF leakage on imaging [1]. Findings at cytochemical analysis of CSF can be normal or reveal increased protein and pleocytosis. The orthostatic headache remits after normalization of CSF pressure [29].

Brain MRI may show subdural fluid collections, descent of the midbrain and cerebellar tonsils, a reduction in the size of the prepontine cistern, dilation of the dural sinuses and spinal epidural plexus, and enlargement of the pituitary gland. However, the most characteristic finding is diffuse pachymeningeal enhancement. Downward displacement of the brain due to low CSF pressure may produce tears in bridging veins in the dural border cell layer, causing them to rupture and leading to subdural hematoma. Downward sagging of the brain produces headache by applying traction to pain-sensitive structures, in particular the VI cranial nerve [2, 3]. The MRI findings in SIH reflect an increase in venous volume throughout the brain. The increased venous volume can be explained by the Monro-Kellie hypothesis in which total intracranial volume is constant and volume equilibrium among its constituents (brain tissue, CSF, and blood) means that a decrease in the volume of one constituent must be compensated by an increase in another constituent [30]. Nevertheless, any or even all of these imaging features may be absent in patients with SIH.

CVT has occasionally been observed in patients with SIH. The number of observations, although limited, seems to indicate that the association between these two entities cannot be simply interpreted as a coincidental finding. We found 26 reports totaling 35 patients with both CVT and SIH [4–28], to which we add two cases. Although an association between SIH and CVT was not reported until 2004 [4], the development of CVT following an iatrogenic spinal CSF leak had been noted since the 1980s [30, 31].

A review published in 2008 [17] noted that SIH is a risk factor for CVT, but CVT is found in only 2% of patients with SIH. As we have seen in our cases, the presence or development of CVT in SIH may be associated with little or no change in the characteristics of the headache [17].

Several mechanisms can explain how SIH can lead to CVT. First, as dictated by the Monro-Kelly Doctrine, in a closed compartment such as the intracranial and spinal dural space, any loss of one component must be compensated by an increase in another one. Therefore, the CSF volume that is lost must be replaced by an increase in the most easily expansible component, which is venous blood [32]. Venous engorgement causes both the appearance of a thickened dura and a relative accumulation of contrast material. The dilation of cerebral veins and sinuses results in a decrease in blood flow velocity. Transcranial Doppler ultrasound has demonstrated that blood flow velocities in the straight sinus decrease by about 47% after lumbar puncture. As patients with SIH probably lose more CSF volume than those undergoing lumbar puncture, it is reasonable to suppose that their decrease in blood flow velocity is even more marked [33].

Second, SIH is associated with rostrocaudal sagging of the brain due to the loss of CSF buoyancy [3], resulting in a negative intracranial pressure gradient that may damage the venous endothelial lining by stretching the cerebral vessels [17]. Third, the loss of CSF reduces absorption of CSF into the cerebral venous sinuses, resulting in increased blood viscosity in the venous compartment [34], which could contribute to dural sinus thrombosis in patients with risk factors for thrombosis, such as hereditary thrombophilia (mainly the factor V Leiden mutation (15%–17% of cases) and the prothrombin-gene-mutation 20210GA (10%–12% of cases); by contrast, antithrombin III-, protein C-, and protein S-deficiency are found in only 2%–6% of cases), anti-cardiolipin antibodies, hyperhomocysteinemia, cesarean delivery, pregnancy-related hypertension, and the use of oral contraceptives.

The occurrence of intracranial hypotension in patients with CVT raises difficult practical questions about the treatment of the two conditions. The first-line treatment for intracranial hypotension is an epidural blood patch, but it is unknown whether early blood patch treatment would avoid the occurrence of venous thrombosis. On the other hand, there is now a consensus that the treatment for CVT should be heparin since a meta-analysis of the only two randomized studies concluded heparin treatment is safe and is associated with a clinically relevant (though not statistically significant) reduction in the risk of death and dependency. Nevertheless, the potential benefit of anticoagulation must be weighed against the risk of subdural hematoma, especially when subdural fluid collections are present [20, 35].

In most reported patients, SIH was treated conservatively and CVT was treated with anticoagulation (Table 1). However, we advocate aggressive treatment of the underlying spinal CSF leak, particularly when symptoms of SIH persist.

## Figures and Tables

**Table 1:** Clinical and radiological data on 33 patients with cerebral venous thrombosis and spontaneous intracranial hypotension.

Authors, year	Age-sex	MRI SIH	MRI CVT	CVT location	OP	Location of CSF leak	AC	EBP	Complications
Berroir et al., 2004 [4]	46 F	Yes	Yes	SSS, RLS	5	?	Yes	No	
	32 F	Yes	Yes	SSS, RLS	17	?	Yes	No	
Sopelana et al., 2004 [5]	56 M	Yes	Yes	Right TS, SS, JV, and SSS	?	?	Yes	No	
Flemming and Link, 2005 [6]	31 F	Yes	Yes	SSS and Right TS	3	?	Yes	No	Dural AV fistula
Savoiardo et al., 2006 [7]	31 M	Yes	Yes	SSS, left TS, and SS	?	Cerv/Thor	Yes	No	
	40 M	Yes	Yes	SSS, StS, and the initial segment of both TS	?	?	Yes	No	Venous infarct
Lai et al., 2007 [8]	45 F	Yes	Yes	Left frontal CV	1	?	Yes	Yes	Venous infarct, seizure
Kataoka et al., 2007 [9]	36 M	Yes	Yes	SSS	4	Cerv/Thor	Yes	Yes	Subdural hemorrhage
Albayram et al., 2007 [10]	45 M	Yes	Yes	SSS	?	Thoracic	Yes	Yes	
Lan et al., 2007 [11]	36 M	Yes	Yes	CV (right vein of Trolard)	?	?	No	No	Seizure, intracerebral hemorrhage, Dural AV fistula, subdural hematoma
Richard et al., 2007 [12]	38 M	Yes	Yes	SSS, both TS and bilateral parietal CV	?	?	Yes	No	
	60 F	Yes	Yes	Right parietal CV	?	?	Yes	No	Right parietal hematoma, hemiplegia
Wang et al., 2007 [13]	33 F	Yes	Yes	CV (left vein of Trolard, vein of Labbé)	8	Cervical	No	Yes	Seizure
Takeuchi et al., 2007 [14]	32 M	Yes	Yes	SSS and CV (vein of Labbé)	5	?	No	Yes	
Tan et al., 2008 [15]	46 F	Yes	Yes	Left TS and SS	?	?	Yes	No	
	40 M	Yes	Yes	SSS and left hemispheric CV	?	?	Yes	No	
Schievink and Maya, 2008 [16]	26 F	Yes	Yes	Left TS and SS	5	Thoracic	Yes	Yes	
	32 M	Yes	Yes	SSS, TS, and cortical veins	0	Thoracic	Yes	Yes	
	43 M	Yes	Yes	SSS, right TS, and SS	4	Thoracic	Yes	Yes	Venous infarct, seizure, transient diplopia
Haritanti et al., 2009 [17]	42 M	Yes	Yes	SSS and right TS	2	?	Yes	No	Seizure, intracerebral hemorrhage
Seiler and Hamann, 2009 [18]	48 F	Yes	Yes	SSS	?	?	Yes	No	Epileptic seizure
Ivanidze et al., 2010 [19]	33 F	Yes	Yes	SSS	?	?	Yes	No	
Nardone et al., 2010 [20]	44 M	Yes	Yes	SSS and CV on the right side	4	?	Yes	No	Subdural hemorrhage
Yoon et al., 2011 [21]	26 M	Yes	No	SSS	5	Cervical	No	Yes	
Dangra et al., 2011 [22]	35 M	Yes	Yes	SSS and SS	?	Cervical	Yes	No	Subdural hemorrhage
Mao et al., 2011 [23]	34 M	Yes	Yes	SSS, ISS, and StS	0	?	Yes	No	Subdural hemorrhage
Ferrante et al., 2012 [24]	59 M	Yes	Yes	SSS and frontal CV	32	?	Yes	No	
Tian and Pu, 2012 [25]	41 F	Yes	Yes	SSS, both sides of the TS and SS	3	?	Yes	No	
Costa et al., 2012 [26]	48 F	Yes	Yes	Left TS	?	?	Yes	No	Transient diplopia, blurred vision
Rozen, 2013 [27]	?	Yes	Yes	SSS, TS, SS, and proximal JV	?	?	Yes	No	Probable seizure
Rice et al., 2013 [28]	75 M	Yes	Yes	SSS	?	?	Yes	No	Status epilepticus, intracerebral hemorrhage
Present study	29 F	No	Yes	SSS and multiple CV	3	Thoracic	Yes	Yes	
	54 M	Yes	No	CV (right vein of Trolard)	2	Thoracic	Yes	Yes	Subarachnoid hemorrhage, right parietal hematoma

SIH = spontaneous intracranial hypotension, MRI = magnetic resonance imaging, CVT = cerebral venous thrombosis, OP = opening pressure (cm H_2_O), CSF = cerebrospinal fluid, AC = anticoagulation, EBP = epidural blood patch, and AV = arteriovenous.

Location: SSS = superior sagittal sinus, RLS = right lateral sinus, ISS = inferior sagittal sinus, TS = transverse sinus, SS = sigmoid sinus, StS = straight sinus, CV = cortical veins, JV = jugular vein.
